# Effect of Steel Fibers on Tensile Properties of Ultra-High-Performance Concrete: A Review

**DOI:** 10.3390/ma17051108

**Published:** 2024-02-28

**Authors:** Wanghui Du, Feng Yu, Liangsheng Qiu, Yixuan Guo, Jialiang Wang, Baoguo Han

**Affiliations:** 1 School of Civil Engineering, Dalian University of Technology, Dalian 116024, China; dwh755981@163.com (W.D.); qiuls@mail.dlut.edu.cn (L.Q.); guoyx@mail.dlut.edu.cn (Y.G.); 2 Department of Civil and Architectural Engineering, Aarhus University, 8000 Aarhus, Denmark; jlwang@cae.au.dk

**Keywords:** UHPC, tensile strength, steel fiber, fiber content, hybrid fiber, digital image correlation

## Abstract

Ultra-high-performance concrete (UHPC) is an advanced cement-based material with excellent mechanical properties and durability. However, with the improvement of UHPC’s compressive properties, its insufficient tensile properties have gradually attracted attention. This paper reviews the tensile properties of steel fibers in UHPC. The purpose is to summarize the existing research and to provide guidance for future research. The relevant papers were retrieved through three commonly used experimental methods for UHPC tensile properties (the direct tensile test, flexural test, and splitting test), and classified according to the content, length, type, and combination of the steel fibers. The results show that the direct tensile test can better reflect the true tensile strength of UHPC materials. The tensile properties of UHPC are not only related to the content, shape, length, and hybrids of the steel fibers, but also to the composition of the UHPC matrix, the orientation of the fibers, and the geometric dimensions of the specimen. The improvement of the tensile properties of the steel fiber combinations depends on the effectiveness of the synergy between the fibers. Additionally, digital image correlation (DIC) technology is mainly used for crack propagation in UHPC. The analysis of the post-crack phase of UHPC is facilitated. Theoretical models and empirical formulas for tensile properties can further deepen the understanding of UHPC tensile properties and provide suggestions for future research.

## 1. Introduction

Ultra-high-performance concrete (UHPC) is one of the latest advances in concrete technology and is a new concrete series [1]. Compared to conventional concrete (CC), UHPC shows superior mechanical properties, durability, and ductility after cracking [2,3,4,5]. At present, there is no widely accepted definition of UHPC, but it is generally considered that its compressive strength is ≥150 MPa and its tensile strength after cracking is ≥5 MPa [6,7]. Under the same bearing capacity, the weight of a structure made of UHPC is only 1/3~1/2 that of CC [8]. Therefore, UHPC is often used in long-span structures and high-rise buildings. In addition, UHPC is, furthermore, energy efficient and environmentally friendly when considering the entire life cycle of the building [6].

The tensile strength is an important factor that affects the overall performance of concrete structures, and it is, therefore, crucial to have the correct material input in the design calculations [9]. In some projects, structural damage is often caused by an insufficient tensile strength and compression zones that have not reached their bearing limits. Steel fibers are crucial for improving the tensile properties of UHPC, so it is important to investigate the effect of steel fibers on the tensile strength of UHPC. Usually, the common methods to improve the tensile properties of UHPC by steel fiber are as follows [10,11,12,13]: (a) increase the fiber content; (b) use different types of fibers; (c) increase the fiber length; (d) hybrid of different types of fibers. However, due to the complexity of the fiber geometry and the uncertainty of the fiber distribution direction, the tensile properties of UHPC materials vary greatly [14,15,16,17,18]. At the same time, the physical properties of the steel fibers and the bonding strength between the fibers and the UHPC matrix also affect the mechanical properties of the UHPC [17,19,20]. This is also a core factor to be considered in structural design. In addition, the steel fibers are also the main reason for the high cost and carbon footprint of UHPC [21]. Therefore, combing and summarizing the knowledge of steel fibers on the tensile properties of UHPC is beneficial for improving the structural design theory of UHPC and reducing the carbon footprint.

With the application of UHPC to engineering practice, the issue of insufficient tensile properties has received increasing attention [22]. By reviewing the latest reviews of UHPC, it was found that the reviews of the effect of steel fiber on the tensile strength of UHPC lacks its influence on the splitting tensile strength [20,21,23,24,25,26,27,28], which must be supplemented. It is very important to detect the cracks in concrete, which is usually carried out by optical means or an extensometer [29]. Digital image correlation (DIC) technology is a non-contact, modern optical measurement experimental technology, which can directly measure the mechanical behavior of materials and structural surfaces. Due to its high measurement accuracy and operation, it has gradually attracted more and more attention in the research on UHPC, especially for the tensile behavior of steel fibers-reinforced UHPC. In this paper, the common test methods for the tensile strength of UHPC, and the influence of the fiber type, fiber length, fiber hybrid, and fiber content on the different testing methods are considered, and the prediction formula and empirical model for tensile correlation are summarized. The application of DIC to UHPC is also introduced. By summarizing and combing the literature, the limitations of previous studies are pointed out, and suggestions for further improvement are put forward to improve the tensile properties of UHPC and promote its wide application.

## 2. Advantages and Significance of Steel Fibers in Concrete

**Figure 1:**
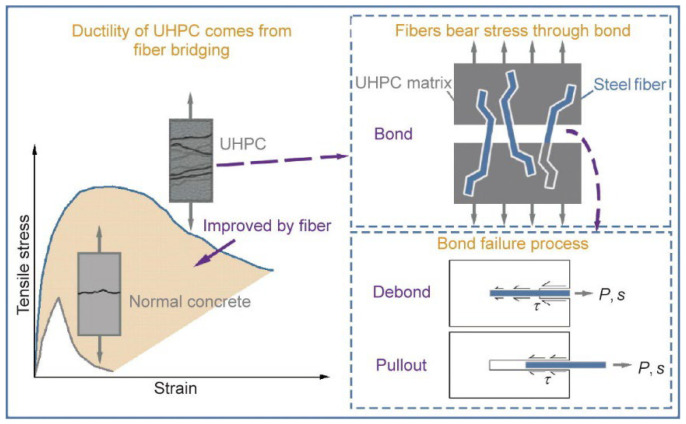
The effect of steel fibers in UHPC compared with normal concrete [36].

Concrete is a quasi-brittle material according to its macroscopic mechanical behavior. As its strength increases, its brittleness increases correspondingly, with the disadvantages of low tensile strength and minor ultimate elongation. To overcome these shortcomings, many researchers have incorporated steel fibers into concrete and have proven its feasibility [30,31,32]. Steel fibers can improve the ductility of the cement matrix by bridging cracks, thus, improving the brittle fracture of concrete [33,34,35]. Usually, adding steel fibers into concrete can play two main roles: (a) improving the tensile strength and (b) preventing the occurrence and propagation of cracks. Figure 1 shows that compared with normal concrete, the tensile strength and toughness of UHPC when steel fibers are added are significantly improved.

**Figure 2:**
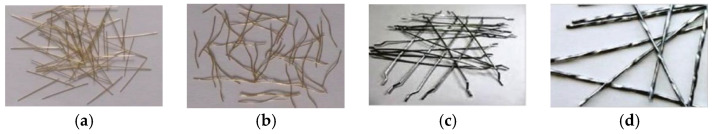
Common types of steel fibers. (**a**) Straight; (**b**) corrugated; (**c**) hooked-end; (**d**) twisted.

Steel fiber is one of the most commonly used fibers in UHPC. Those properties related to the tensile strength of a UHPC depend largely on the content and geometric size of the steel fiber [21,37]. The geometric differences among (in Figure 2) and fiber content (vol.%) of steel fibers have been discussed in many studies [38,39]. The motivation for this kind of research is that steel fiber makes an important contribution to the tensile properties, and steel fiber is one of the most expensive materials in UHPC [21]. Therefore, discussing the influence of steel fiber on UHPC is beneficial for improving the tensile strength of UHPC, reducing the cost of UHPC, and making it more suitable for engineering practice.

**Figure 3:**
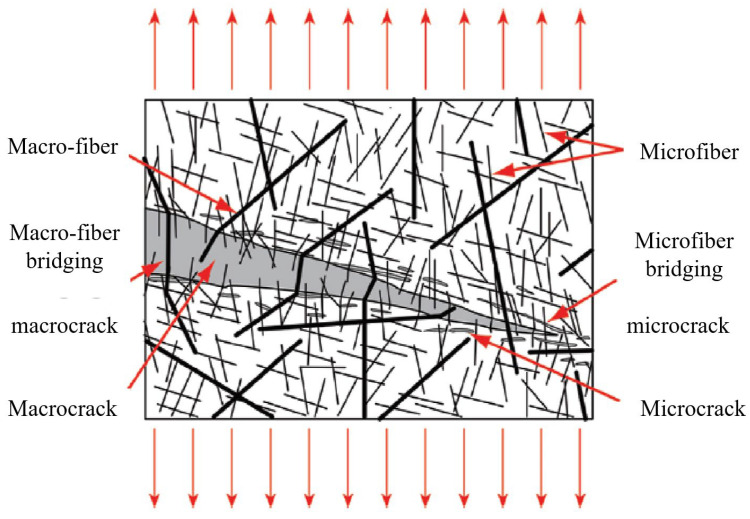
Schematic diagram of fiber bridging effect of fiber hybrid [20].

According to previous experience, steel fibers can be divided into micro steel fibers ($l_{f}$/$d_{f}$ ≤ 13/0.2) and macro steel fibers ($l_{f}$/$d_{f}$ > 13/0.2). Based on their length, they can also be divided into short steel fibers (6 mm < $l_{f}$ < 13 mm), medium–long steel fibers (13 mm ≤ $l_{f}$ ≤ 20 mm), long steel fibers (20 mm < $l_{f}$ < 30 mm), and ultra-long steel fibers (30 mm ≤ $l_{f}$). It is generally believed that a reasonable hybrid of two or more kinds of steel fibers can be added to a UHPC, which can not only play up the advantages of the different fibers, but also reflects their synergistic effect, which can significantly improve some properties of the UHPC, and reduce costs. Figure 3 shows the synergy between macro and micro steel fibers.

## 3. Research Methods

As mentioned above, although the definition of UHPC materials in the world has not been completely unified, there is a certain consensus on its matrix compactness and compressive strength (e.g., compressive strength ≥ 150 MPa, excellent durability, etc.). Under these consensus conditions, this paper focuses on the following issues:

(1) What experimental methods or standards are used to study the tensile properties of UHPC?
(2) What are the effects of different fiber contents, shape, and hybrids on the tensile properties of UHPC?

**Figure 4:**
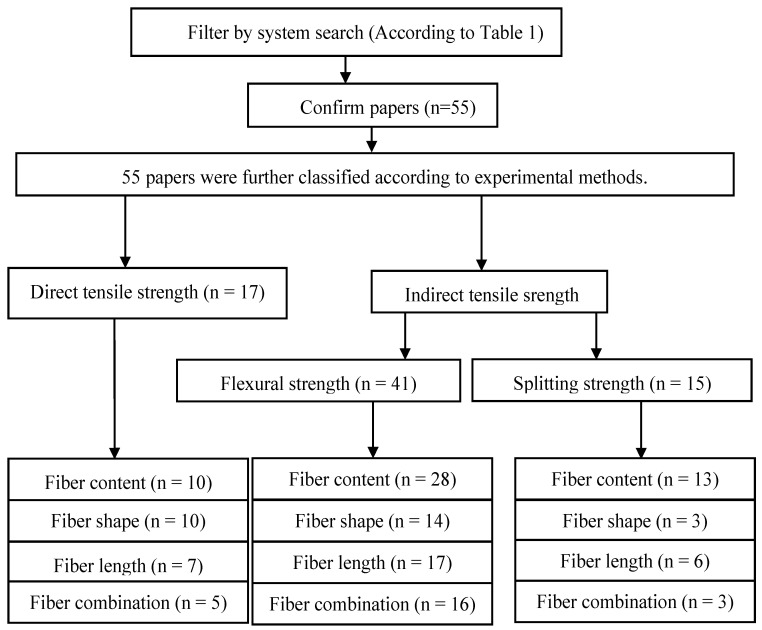
Flow chart.

For the above problems, we identified three experimental methods, namely, the direct tensile test, flexural test, and splitting test, which are often used in UHPC tensile strength tests [40]. The relevant research papers were found through a systematic literature search of the “Web of Science”. Such databases are widely used in engineering research fields. Figure 4 shows the preliminary screening results of the papers that needed attention in this study based on “steel fiber”, “UHPC”, and “paper abstract”. The specific details are shown in Table 1. Finally, the collected papers are summarized in Table 2 according to the experimental methods and fiber properties. In the subsequent analysis, we classified the type and size of the specimens, and the loading methods and testing standards used in the different experimental methods to provide guidance for future tensile property testing of UHPC. At the same time, the first-cracking strength and tensile strength of the steel fibers added to the UHPC were considered. To more intuitively observe the effects of the fiber and specimen geometry, we extracted the tensile strength results for the UHPC for 28 days. The stress–strain curve of the direct tensile strength test and the load–deflection curve of the flexural strength test were further analyzed. In order to enrich the design model for UHPC, we summarized the empirical formulas and prediction models for the three experimental methods. In addition, DIC, as a new technique, has been widely used in the study of UHPC tensile crack growth. It is necessary to understand its application process to UHPC, which is of great significance for studying the tensile softening part of UHPC.

## 4. Results and Discussion

Table 2 summarizes the UHPC test performance and steel fiber information; the commonly used steel fiber content is 0~3 vol.% when studying the tensile properties, and the ultimate fiber tensile strength is above 2000 MPa. This is to avoid the brittle fracture of the UHPC when subjected to tension. The tensile property testing method is similar to that of CC, that is, direct tension and indirect tension (flexural strength and splitting). In terms of the fiber types, straight steel fibers are the most commonly used. Even when selecting deformed steel fiber, straight steel fiber is often used as the reference. This may be because the production process for straight steel fiber is simpler than that for deformed steel fiber. From the collected papers, straight steel fibers ($l_{f}$/$d_{f}$ = 13/0.2), hooked-end steel fibers ($l_{f}$/$d_{f}$ = 30/0.38), and twisted steel fibers ($l_{f}$/$d_{f}$ = 30/0.3) are the most commonly used sizes. There are only two articles and one article on the effects of corrugated ($l_{f}$/$d_{f}$ = 13/0.2) [59,61] and spiral ($l_{f}$/$d_{f}$ = 13/0.2) [48] steel fibers on tensile strength, respectively, and the sizes of the commonly used corrugated steel fibers and spiral steel fibers cannot be analyzed.

### 4.1. Effect of Steel Fibers on Direct Tension Strength

Direct tensile test can show the tensile strength and strain hardening/softening behavior of UHPC more intuitively. It can directly provide the stress data for structural calculations without the need for back-analysis, like other tensile methods [87]. However, the traditional method of arranging the strain gauges to measure the direct tensile strength often makes it difficult to obtain effective data because of the limited arrangement area of the strain gauges. This also means that new nondestructive testing techniques are needed to study the cracking process of UHPC, in order to gain a more comprehensive understanding of the direct tensile cracking process of UHPC. At the same time, direct tension test usually requires more experimental samples (compared with flexural tensile test and splitting tensile test). This because it is often difficult to ensure that the sample is not eccentric when conducting direct tensile test, which leads to the excessive deviation of the results. Therefore, when conducting direct tensile experiments, it is often necessary to conduct experiments on more specimens with the same fiber content to avoid the probability of excessive discreteness in the experimental results.

#### 4.1.1. Test Setup for Direct Tensile Strength

**Figure 5:**
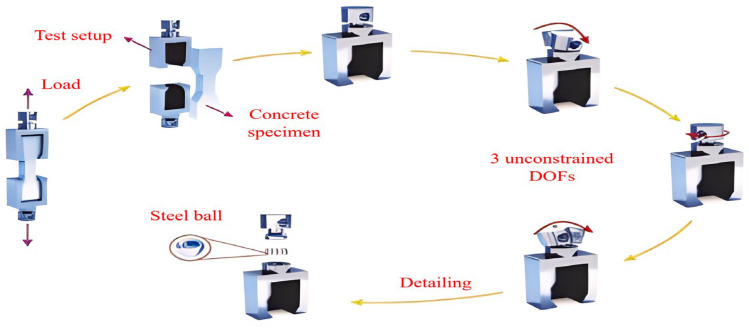
Direct tension test details and end-restraint conditions [84].

Use a testing machine with the appropriate range, pre-loaded to the force value of 60~100 N to ensure that the sample is not eccentric, and the elongation of the tensile sample is obtained by two LVDTs (Linear Variable Displacement Transducer) on the frame fixed in advance on both sides. Displacement control is commonly used during the loading process, and the displacement rate is 0.05~0.6 mm/min. The samples used for the direct tensile strength of the UHPC are dog-bone shaped and prism shaped, of which the dog-bone shape is the most commonly used, and most tests are not carried out according to the relevant standards (in Table 3) [95]. Some researchers refer to the testing methods of cement mortar standard AASHTOT 132–87 [96], and common concrete standards GB/T50081-2019 [97] and FHWA [98], and some use the special standards for UHPC, such as JSCE [99], T/CBMF37-2018 [100], and NFP18-710 [101]. It is found that even the special standards for UHPC need to refer to the standards for ordinary concrete [75]. The number of researchers who have conducted direct tensile testing of UHPC has been limited by the difficulty of obtaining a uniformly distributed stress and a stable crack opening response across the cross section of the dog-bone specimens. However, the type of direct tension mode also has a large influence on the experimental results. Usually, pinned-end conditions are beneficial for specimen alignment and uniform stress distribution before cracks, but compared with fixed boundary conditions, it does not support uniform crack opening in the whole cross section [57]. Figure 5 shows that the tensile test method only constrains the axial degree of freedom.

#### 4.1.2. Effect of Steel Fiber Content and Shape on Direct Tensile Strength

**Figure 6:**
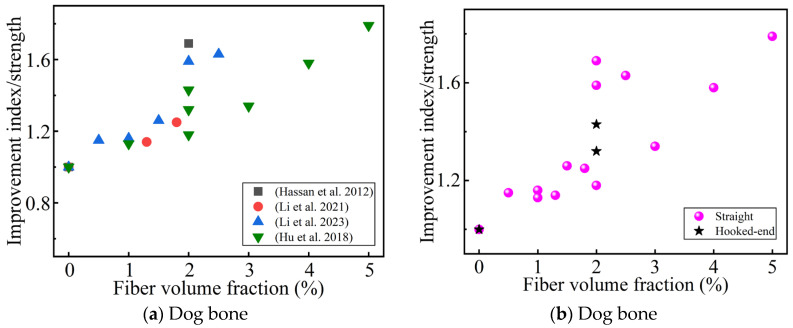
The effect of the steel fibers content and shape on the improvement index of the direct tension strength. (**a**,**b**) Sorted by Ref. and fiber shape, respectively [45,75,80,93].

Figure 6a summarizes the influence of the steel fiber content on the improvement index of the direct tensile strength. All the studies agree that the direct tensile strength increases with an increase in the steel fiber content (up to 5 vol.% [88,93]), even reaching 3.2 times that of a UHPC without fiber reinforcement [79]. The size of the dog-bone specimen affects the improvement of the direct tensile strength by the fiber content [45,75,80,93]. The fiber content affects the strain-hardening phenomenon of the UHPC. Some researchers believe that the strain-hardening phenomenon occurs when the fiber content reaches 1.5 vol.% or above [40,57,64]. However, Liu et al. [48] studied a UHPC with a coarse aggregate, and found that even when the steel fiber content was as high as 2.5 vol.%, no strain-hardening phenomenon was observed in the matrix. This may have been because the addition of the coarse aggregate damaged the microstructure of the UHPC. When the fiber content is 2%, hooked-end steel fibers seem to improve the direct tensile strength better than straight steel fibers (in Figure 6b), which is related to the mechanical anchoring of the hooked-end steel fibers.

**Figure 7:**
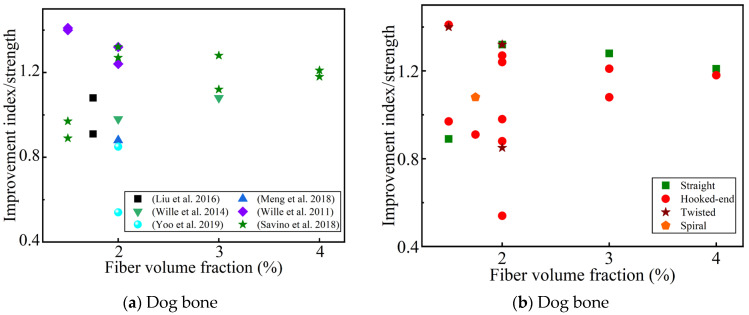
The effect of the fiber content and shape on the improvement index of the direct tensile strength. (**a**,**b**) Sorted by Ref. and fiber shape, respectively [48,50,57,58,64,88].

Figure 7 summarizes the influence of the fiber content and shape on the improvement index of the direct tensile strength relative to the addition of micro-straight fibers ($l_{f}$/$d_{f}$ = 13/0.2) to the UHPC. At high fiber contents (2 vol.%), the hooked-end fibers always seem to improve the direct tensile strength better than microscopic straight fibers (in Figure 7b). This may be due to the large size of the hooked-end steel fibers selected and the lower number of fibers when the fiber content is low. It seems that the twisted and straight fibers are always better than the hooked-end fibers regarding the first-cracking strength [43,52,57,58]. Studies have shown that adding deformed steel fibers is more conducive to improving the first-cracking strength than adding micro-straight fibers [48]. All these indicate that the addition of steel fiber is conducive to the improvement of the first-cracking strength of UHPC. However, adding deformed steel fibers to UHPC is not necessarily beneficial for the improvement of the direct tensile strength [43,50,64]. One possible explanation for this is that the mechanical anchoring and congestion of the fibers causes damage to the UHPC matrix [64]. Therefore, the improvement of the tensile properties of UHPC using steel fibers depends not only on the shape of the fibers, but also on their length and content [48].

#### 4.1.3. Effect of Steel Fiber Length and Hybrids on Direct Tensile Strength

Some studies have found that hooked-end fibers with a fiber length of 13~30 mm do not significantly improve the direct tensile strength [64,79]. Park et al. [52] also obtained similar findings when studying the effect of ultra-long hooked-end fibers on the direct tensile strength of UHPC. What these studies have in common is that short steel fibers are always in a slightly higher direct tension than long fibers. LeHoang et al. [40] found that straight fibers with a length of 13 mm are always superior to short fibers ($l_{f}$ = 9 mm) and medium–long fibers ($l_{f}$ = 20 mm) in both their direct tensile strength and first-cracking strength. Liu et al. [48] also obtained similar findings when studying hooked-end steel fibers of different lengths. Savino et al. [88] found that the effect of fiber length on the direct tensile strength varied with fiber content under the same fiber content. In summary, fiber lengths of 13~20 mm seem to be more conducive to the improvement of the direct tensile strength.

It is generally believed that micro steel fibers can delay and prevent the development of microcracks, while macro steel fibers can delay the expansion of large cracks and the damage of the composite materials [75,80]. Therefore, the appropriate combination of the two can improve the performance of UHPC. In the study of the direct tensile properties of hybrid steel fibers on UHPC, some researchers believe that replacing ultra-long deformed fibers with microfibers is more conducive for improving the direct tensile strength [43,52]. In the research on the tensile properties of UHPC which combine straight and deformed steel fibers, some researchers think that the higher the content of straight steel fibers, the better the effect [50], while others think that a ratio of 1:1 is the best for improving the direct tensile strength [84]. In the study of the tensile properties of UHPC with different lengths of deformed steel fibers, some researchers have found that the combination effect was not as good as that of a single, straight steel fiber [93]. These results show that the effectiveness of the synergistic effect of different types and lengths of steel fiber combinations is the key to improving the direct tensile strength.

#### 4.1.4. Stress–Strain Curve and Empirical Formula for Direct Tensile Strength

**Figure 8:**
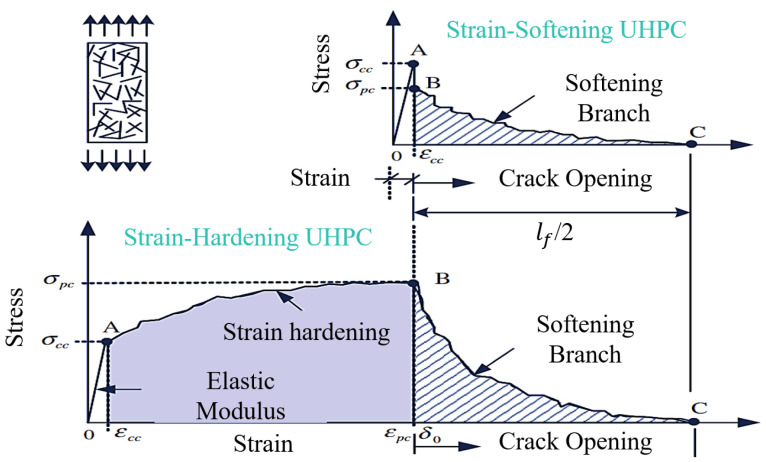
Tensile hardening and softening behavior of typical UHPC [52].

When the steel fiber content is greater than a certain critical value ($V_{f}^{crit}$), the direct tensile strength ($\sigma_{pc}$) of the UHPC is greater than the first-cracking strength ($\sigma_{cc}$), and the UHPC exhibits “strain hardening behavior”; otherwise, it is “strain softening behavior” (Figure 8). The strain-hardening behavior of UHPC can be described in three parts: (a) The elastic stage, whose strength reaches 90~95% of the first-cracking strength, is when crack development and activation occurs. (b) In the strain-hardening stage, the matrix gradually cracks. The cracked part is mainly supported by the steel fibers, which are then transferred to the uncracked matrix. At this stage, there are numerous cracks. (c) In the strain-softening stage, the steel fibers are gradually pulled out to form a larger main crack. For “strain softening behavior”, there is no above-mentioned strain-hardening stage, only the elastic stage and the strain-softening stage. For the tensile stress–strain curve without an obvious first-cracking point, Park et al. [52] determined the first-cracking point by fitting the intersection point of the elastic stage and the strain- hardening stage to reduce its subjectivity. Wille et al. [57] thought that $V_{f}^{crit}$ = 1 vol.%, while Le Hoang et al. [40] and Kay et al. [64] thought that $V_{f}^{crit}$ ≈ 1.5 vol.%, the value of which can be predicted according to the principle of the mixing law [102]:

(1) $$V_{f}^{crit}=\frac{\sigma_{m}}{\eta_{1}\eta_{0}\left(\sigma_{f}-E_{f}\varepsilon_{m}\right)+\sigma_{m}}$$

where $\eta_{1}$ is the fiber length coefficient; $\sigma_{m}$ is the tensile strength of the UHPC matrix; $\eta_{0}$ is the orientation coefficient of the steel fiber, which is 1 for a one-dimensional orientation, 2/π for a two-dimensional random distribution, and 0.5 for a three-dimensional random distribution; $\sigma_{f}$ is the tensile strength of the steel fiber; $\varepsilon_{m}$ is the ultimate strain on the UHPC matrix; and $E_{f}$ is the elastic modulus of the steel fiber.

For the softening stage, the crack width curve is often used to describe it. To evaluate the properties and brittleness of the specimen after cracking, the fracture energy $G_{f}$ and the characteristic length $L_{ch}$ are defined, respectively, and their formulas are as follows [75,80]:

(2) $$G_{f}=\int_{0}^{w_{f}}\sigma \left(w\right)dw$$

(3) $$L_{ch}=\frac{E_{t}G_{f}}{{\left(f_{t}\right)}^{2}}$$

where $E_{t}$ is the modulus of elasticity (GPa), $f_{t}$ is the direct tensile strength (MPa), and the units of $L_{ch}$ and $G_{f}$ are mm and N/m, respectively. The greater the $L_{ch}$, the better the toughness of the UHPC material.

Table 4 summarizes the empirical formula, constitutive model, and prediction formula for the direct tensile strength. Most prediction formulas mainly study a fiber volume fraction of 1.5~3 vol.%, and the fiber shape is straight and hooked steel fiber (as shown in Table 4). Some studies have used the linear tensile model to represent the direct tensile constitutive equation [57], some have used nonlinear models [84], and some have combined linear and nonlinear models to propose three-stage and four-stage constitutive models [79,103,104,105]. These models have a high fit to the pre-strain-hardening part, but a relatively poor fit to the post-strain-hardening part, which depends on the crack growth process of the UHPC. It can be seen that an in-depth understanding of the crack propagation process is important for facilitating the development of UHPC constitutive models. For the prediction of the direct tensile strength, some studies have put forward empirical formulas about the quadratic function of the fiber content, a composite material model considering the fiber orientation, and empirical formulas based on the fiber index [40,57]. In addition, there are simple and efficient models based on the fiber aspect ratio, type, content, fiber-to-matrix density ratio, and matrix strength [88]. These are important for facilitating the wide application of UHPC.

### 4.2. Effect of Steel Fibers on Flexural Strength

As mentioned above, although the direct tensile test can directly provide the tensile behavior (elasticity and strain hardening and softening) of UHPC, it is challenging to implement the direct tensile test. In the structural design of ordinary reinforced concrete, it is generally considered that concrete only bears pressure, but not tension. However, the structural integrity of UHPC is maintained due to the bridging effect of the steel fibers after cracking [19,22]. It is generally believed that there is a certain relationship between the flexural and direct tensile strength. Moreover, the flexural tensile strength itself is also an important evaluation index of flexural performance.

#### 4.2.1. Test Setup for Flexural Tensile Strength

For flexural strength testing, the loading process is usually controlled by displacement. Table 5 summarizes the test methods for flexural strength (three-point and four-point bending tests). The displacement control rate is 0.05~0.6 mm/min. There are also loading methods that are controlled by the researchers [69]. All the specimens selected for flexural strength test are prisms. The calculation of the flexural tensile strength is shown in Equation (17) [84]. Notched beams in three-point bending tests are often used to calculate $G_{f}$ and $L_{ch}$ [44,92]. The calculation formula for $G_{f}$ is shown in Equation (18) [92]. The calculation formula for $L_{ch}$ is similar to the calculation of the direct tensile strength in Equation (3); it is just that $f_{t}$ no longer represents the direct tensile strength but the splitting tensile strength. Since the presence of a notch in the beam will cause stress concentration and early crack initiation from the stress, it is generally considered that notched specimens are not suitable for characterizing the tensile behavior of UHPC [57]. There are different standards for testing the flexural tensile strength of UHPC. The most commonly used standards are the Chinese [107] and European cement standards [49] and ASTMC1609 [108]. In ASTMC1609, the sample size requirement is at least three times the maximum fiber length. Of the collected research papers, all the papers met this requirement, except for some studies on hooked-end steel fibers [44,47,50,73] and straight steel fibers [78,94]. According to previous research reports, the size difference of the samples will affect the distribution of the steel fibers, which will then affect the flexural tensile strength [109,110]. Considering the influence of the size effect and referring to the above commonly used standards, the specimen size is divided into three research objects, namely, a small prism (prism with length = 160 mm, width and depth = 40 mm) and a large prism (prism with length = 350~500 mm, width and depth = 100 mm), and a middle prism between them. In summary, we divided the flexural strength samples of UHPC into a small prism, middle prism, and large prism for further analysis.

(17) $$f_{ff}=\frac{My^{*}}{I}$$

(18) $$G_{f}=\frac{W_{0}+mg\delta }{A}$$

where $f_{ff}$ is the flexural strength (MPa); $y^{*}$ is the farthest point from the neutral axis (mm); *I* is the moment of inertia of the cross-sectional area (mm^4^); *M* is the flexural moment (N·mm); $W_{0}$ is the area under the load–deflection curve (kN·mm); *m*, *g*, δ, and *A* are the mass (g), gravitational acceleration (m/s^2^), deflection (mm), and notch cross-sectional area within the span of the specimen (mm^2^), respectively; the unit for $G_{f}$ is N/mm.

#### 4.2.2. Effect of Steel Fiber Content and Type on Flexural Tensile Strength

**Figure 9:**
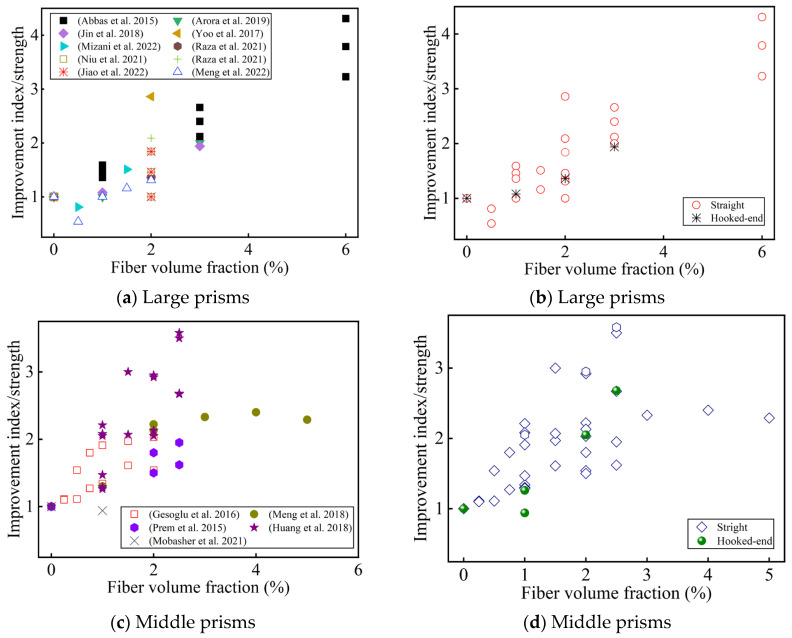

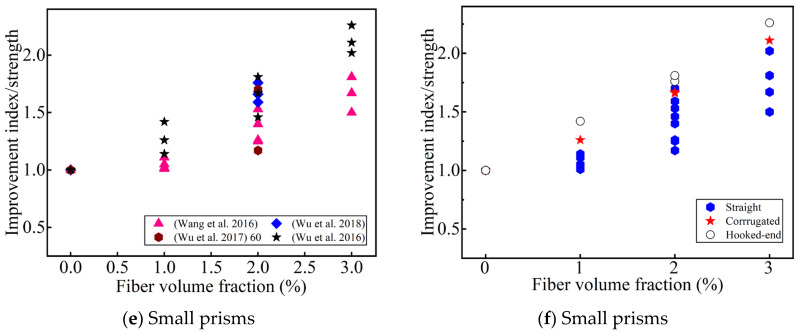
The effect of the fiber content and geometry on the improvement index of the flexural strength. (**a**,**c**,**e**) Sorted by Ref.; (**b**,**d**,**f**) sorted by fiber geometry [41,42,44,46,50,53,56,59,60,61,66,68,69,77,78,81,83,86,91].

**Figure 10:**
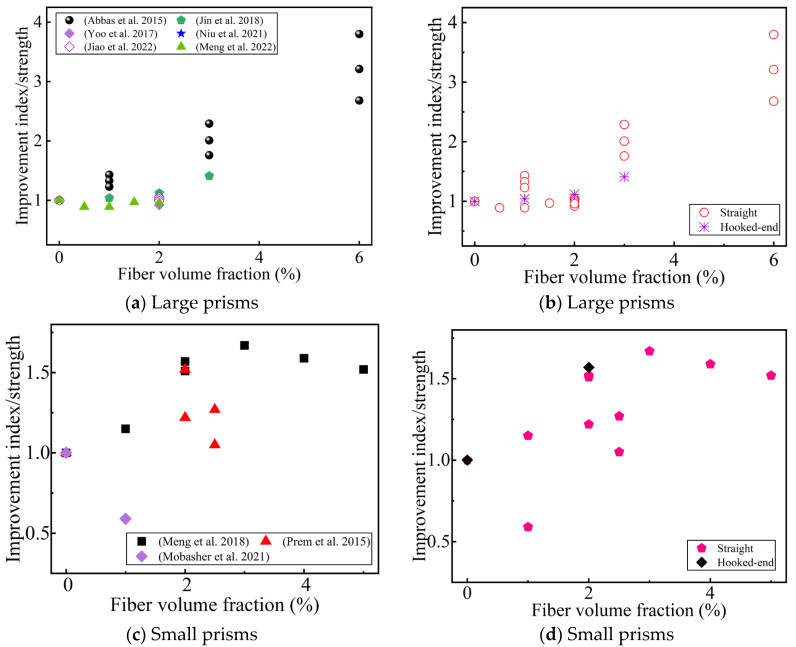
The effect of the fiber content and geometry on the improvement index of the first-cracking strength. (**a**,**c**) Sorted by Ref.; (**b**,**d**) sorted by fiber geometry [41,46,50,53,66,77,81,86,91].

Figure 9 summarizes the effects of the fiber volume fraction and shape on the flexural tensile strength for different specimen sizes compared to a UHPC without fiber reinforcement. Most of the researchers reported that the fiber content improved the flexural strength (even up to 6 vol.%) [121]. This can be attributed to fiber bridging at a high fiber content [41,61]. It is generally believed that when the fiber content reaches a certain value, the flexural strength will decrease as the fiber content increases [44,50]. Meng et al. believed that this value was >3 vol.% [50], adversely affecting the flexural tensile strength due to excess fiber aggregation/balling. Park et al. [51] thought that when the fiber reinforcement index was ${V_{f}l}_{f}$/$d_{f}$ < 0.4, strain-softening behavior would be observed. Some researchers have argued that a low fiber content (<2 vol.%) does not increase the first-cracking strength [51,59,61,66], while a high fiber content (≥2 vol.%) increases the first-cracking strength (in Figure 10). This seems to indicate that adding an appropriate amount of steel fibers to the UHPC can improve the UHPC matrix [51,61]. Different researchers hold different views on the optimal fiber content to improve the first-cracking strength. Meng et al. [50] thought that this optimal value was 3 vol.%. However, Prem et al. [53] thought it was 2 vol.%. There have also been some reports that the fiber content improves the first-cracking strength (up to 6 vol.%) [41]. One possible explanation is that UHPC has a high density and extremely low porosity, and steel fiber bridging reduces the effective crack size [42]. The flexural tensile strength is also affected by the fiber orientation. When the fiber orientation is consistent with the tensile direction, the flexural tensile strength is higher [122,123]. However, the effect of the orientation of the fiber with the first-cracking strength is not obvious, which depends on the compactness of the matrix [91]. Therefore, the orientation of the steel fiber at a high content is also a key to improving flexural performance.

**Figure 11:**
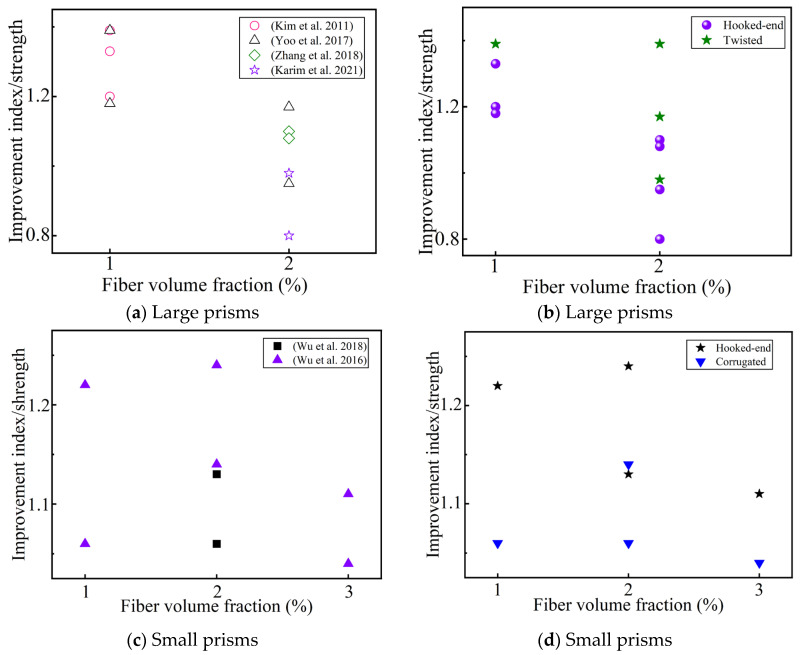
The effect of deformed fibers on the improvement index of the flexural tensile strength, compared with the addition of micro straight fibers to the UHPC ($l_{f}$/$d_{f}$ = 13/0.2). (**a**,**c**) Sorted by Ref.; (**b**,**d**) sorted by fiber geometry [47,59,61,63,67,70].

The reinforcement effect of deformed fiber is better than that of straight fiber in small prisms, but that is not always the case in large prisms (in Figure 11). Deformed steel fibers with a low fiber content (1 vol.%) are superior to micro straight fibers, while deformed steel fibers with a high fiber content (2 vol.%) are not significantly superior to micro straight fibers for improving the flexural tensile strength [63]. This is due to the fact that there are more microfibers with a high fiber content, which was confirmed by Karim et al.’s [70] study on the strength at different deflection points. Khayat et al. [59] believed that the effect of the improvement of the fiber type ($l_{f}$/$d_{f}$ = 13/0.2) on the flexural tensile strength is consistent with the adhesion between the fibers and UHPC matrix; that is, the order of the fiber types based on their effect on the flexural tensile strength is hooked > corrugated > straight steel fiber. However, the effect of the fiber type on toughness shows the opposite trend [61], which shows that the improvement of flexural tensile strength may not effectively improve toughness. In the collected literature, only Gesoglu et al. [44] studied notched prisms using micro straight fibers ($l_{f}$/$d_{f}$ = 6/0.2) as the reference, and believed that macro-fibers effectively improved the flexural tensile strength. Some researchers have studied the influence of fiber shape on the flexural strength after 3 days of steam curing, and have found that fiber types have no significant influence on the flexural tensile strength [49]. However, Al-Osta et al. [73] found that fiber types increase the flexural strength under water and steam curing. This may be due to the mechanical anchoring effect of the deformed steel fibers [49], and the steam curing promoting the hydration of the cementitious materials [73]. Some researchers believe that deformed steel fibers increase the first-cracking strength, and that the curing conditions of the UHPC also have a certain impact on this [73]. Other researchers believe that the first-cracking strength has nothing to do with the shape of the added fibers [61]. In summary, fiber shape, size, the UHPC curing conditions, and fiber content should be considered in a comprehensive manner to understand the influence of fiber type on the UHPC flexural properties.

#### 4.2.3. Effect of Fiber Length and Hybrids on Flexural Tensile Strength

Among the collected papers, it is agreed that fiber length has no effect on the first-cracking strength of UHPC [62,63,74,77,81]. Most studies agree that fiber length improves the flexural tensile strength [53,60,62,63,71,72,74,77,81,94]. One explanation is that long fibers are less likely to be pulled out of the matrix, thus, increasing the flexural tensile strength. This has been confirmed by Yoo et al.’s [62] research, and they also reported that long fibers form more microcracks and have a lower average crack spacing. Some studies have found that at a low fiber content (≤1 vol.%), the fiber length increases the flexural tensile strength [51,65]. However, the opposite trend has been found when the fiber content was >1 vol.% [65,66]. This seems to indicate that the stronger interactions between longer fibers are detrimental to fiber alignment [51,65]. Huang et al. [71,72] used a special L-shaped mold to cast a UHPC, and found that the long fibers were blocked at a high content, which indicates that the mutual interference between a large number of long fibers affects the flexural tensile strength. However, Abbas et al. [41] found that a UHPC with short fibers ($l_{f}$ = 8 mm) has the highest flexural strength and a smaller crack width. Therefore, understanding the crack propagation law of UHPC is of great significance to improving its flexural strength.

Regarding the effect of straight fiber hybrids on the flexural strength, most researchers believe that the first-cracking strength is not affected by fiber hybrids [47,55,66,74,77,81]. Kim et al. [47] kept the content of the macro steel fiber ($l_{f}$/$d_{f}$ = 30/0.3) at 1 vol.%, and then added 0.5~1.5 vol.% of micro steel fiber ($l_{f}$/$d_{f}$ = 13/0.2), and found that the higher the microfiber content, the higher the flexural strength. However, some researchers have found the opposite trend [60,66,77,81]. This is because macro-fibers play a decisive role in the strength enhancement after microcracks [60,66]. In addition, it seems that the flexural tensile strength is the highest when the content of macro-fibers is maintained at 1.5 vol.% and the content of microfibers is 0.5~1 vol.% [60,66,74,77,81]. One explanation is that the presence of microfibers facilitates the fiber arrangement [66,74]. Ryu et al. [55] found that the improvement in the flexural strength of medium–long fibers and long fiber hybrids were better than that of single fibers. This shows, to a certain extent, that fiber hybrids provide an idea for improving the flexural tensile strength while reducing the fiber content.

In the collected papers, all the researchers have consistently believed that a hybrid of straight and deformed steel fibers does not improve the first-cracking strength [47,50,53,63,67,70,73]. Yoo et al. [71] found that the improvement in the flexural tensile strength after the hybridization of medium–long straight fibers ($l_{f}$ = 19.5 mm) and long deformed fibers ($l_{f}$ = 30 mm) was inferior to that of a single medium–long straight fiber. Kim et al. [47] found that hybrid, long deformed fibers ($l_{f}$ ≥ 30 mm) and medium–long straight fibers ($l_{f}$ = 13 mm) improve the flexural strength, which is related to the content of the medium–long fibers. Some researchers have found that the ratio of the medium–long straight fibers ($l_{f}$ = 13 mm) to the long deformed fibers ($l_{f}$ ≥ 20 mm) is 1:1 when the flexural tensile strength is the highest [50,54,67,70]. Similar findings were also found by Ma et al. [49] after 3 days of steam curing. This seems to indicate that the optimal flexural tensile strength can be obtained when the hybrid of medium–long straight fibers and long deformed fibers is 1:1. However, Al-Osta et al. [73] found that the improvement in the flexural tensile strength obtained by mixing long-hooked-end fibers and medium–long straight steel fibers under water curing and steam curing was positively correlated with the content of the hooked-end fibers. In summary, the synergistic effect between straight fibers and deformed fibers has a great influence on the flexural tensile strength, which needs further study.

#### 4.2.4. Load–Deflection Curve and Empirical Formula

**Figure 12:**
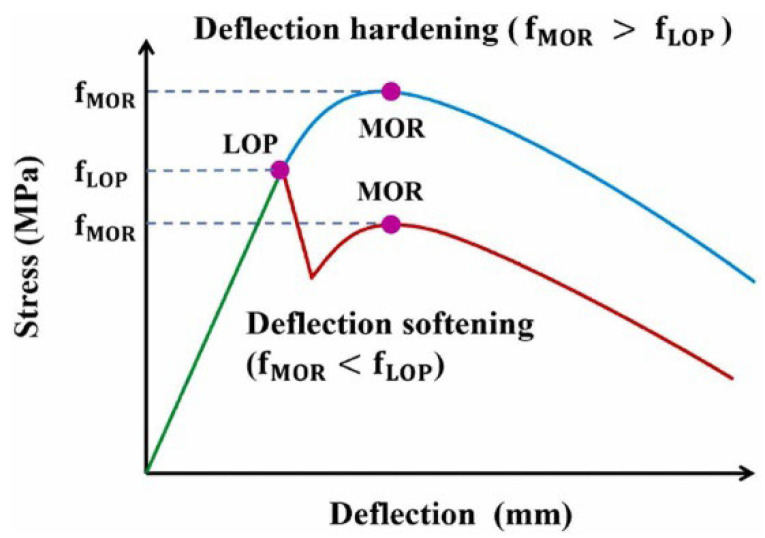
Typical load–deflection curve [81].

Previous research has determined the load–deflection curve of UHPC, as shown in Figure 12. According to the rise and fall of the curve after the linear stage, it is divided into deflection-hardening behavior and deflection-softening behavior. The linear elastic turning point of the load–deflection curve is defined as the limit of proportionality (LOP) [81]. The nonlinear maximum point of the load–deflection curve is defined as the modulus of rupture (MOR). Some scholars have defined the maximum stress of flexural-softening behavior as the MOR [84]. Its constitutive model can be expressed by the following formula [61,81,84]:

(19) $$y=\frac{ax+bx^{2}}{1+cx+dx^{2}}$$

However, the parameters of the above formula are determined by the fitting experimental results, which is more complicated and has an unclear physical meaning. Wu et al. [61]. suggested that the UHPC load–deflection curve be modeled in two sections.

The ascending part is as follows:

(20) $$y=\frac{a_{1}x-x^{2}}{1+\left(a_{1}-2\right)x}$$

where y and x are the load and deflection ratio coefficients, respectively. $y=F/F_{peak}$; $x=\delta /\delta_{peak}$; and $a_{1}$ and $b_{1}$ are obtained by least square fitting, $a_{1}$ ≥ 1, $b_{1}$ ≥ 0.

Table 6 summarizes the empirical prediction formulas for the flexural strength. Some researchers have compared the relevant standards JG/T472-2015 [124], ACI318-95 [125], and ACI363R-92 [126] through their experiments, and found that JG/T472-2015 overestimates the flexural tensile strength of UHPC, while ACI363R-92 seems to be able to predict the flexural strength with a fiber volume fraction ≤2 vol.%, but the data amount is small and needs further confirmation [46]. Some researchers have used composite material theory to predict the flexural strength of a UHPC with straight fibers, and found that the results were quite different from the experimental results [59]. Some researchers have used an L-shaped-induced flow-induced device and the composite material model to propose a prediction model for the flexural strength of a UHPC with straight fibers of different lengths [72]. It has also been recommended that in order to ensure the maximum flexural tensile strength, the ratio of the fiber length to the horizontal outlet height ($l_{f}$/H) should be greater than one [71]. In addition, some have put forward an empirical formula for the quadratic equation between the flexural tensile strength and fiber content [82]; some have put forward an empirical formula for b/w, the fiber comprehensive coefficient [83] and the fiber reinforcement index [66]; and some have put forward an empirical formula for the flexural tensile strength of straight and hooked-end fibers [73]. In summary, at present, the flexural tensile strength models of UHPC are mostly based on single steel fibers, and the hybrid prediction models of different shapes of steel fibers still need further study.

### 4.3. Effect of Steel Fibers on Splitting Tensile Strength

Compared with the 41 papers in Table 5 on UHPC flexural strength, only 15 papers, in Table 7, have studied the splitting tensile strength of UHPC. This is because the splitting test is generally more suitable for CC, where cracks will fail immediately. However, direct tensile testing is difficult, and flexural tensile testing results may show the measured tensile strength as being greater than the true tensile strength of the material, due to size effects and interface stress gradient effects. Therefore, the splitting strength test is a commonly used indirect method with which to test the tensile strength of UHPC. It is helpful to evaluate the stability of the UHPC structure under splitting, which has practical engineering significance.

#### 4.3.1. Test Setup for Splitting Tensile Strength

For splitting tensile strength testing, the loading process is usually controlled by displacement/force. From Table 7, it is known that the geometric shapes of the specimens used for the splitting strength are the cylinder and prism, in which the ratio of the diameter to the height of the commonly used cylinder specimens is 1:2 (D:H = 1:2); the commonly used prism is a cube with a side length of 100 mm. The calculation formula for the splitting tensile strength is shown in Equation (29). According to different countries and regions, both geometric shapes are commonly used, and the test standard ATSMC496 [127] is commonly used.

(29) $$f_{ft}=\frac{2P}{\pi A}$$

where $f_{ft}$ is the splitting tensile strength (MPa), P is the failure force (N), and A is the area of the splitting face (mm^2^).

#### 4.3.2. Effect of Fiber Content and Fiber Type on Splitting Tensile Strength

**Figure 13:**
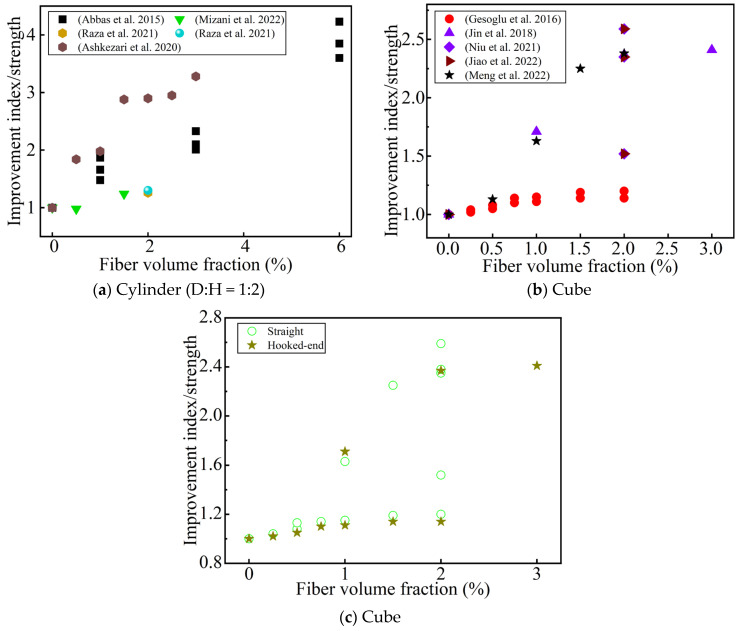
The effect of the steel fibers’ content and geometry on the improvement index of the splitting tensile strength. (**a**,**b**) Sorted by Ref.; (**c**) sorted by fiber type [41,44,46,68,69,77,78,81,82,86].

Figure 13 summarizes the improvement index of the fiber content and geometry on the splitting strength of UHPC compared with that of non-fiber-reinforced UHPC. Most studies have found that the splitting tensile strength is positively correlated with the fiber content (up to 6 vol.%) [41]. Some researchers have also found that when the fiber content is 2 vol.%, the direct tensile strength of a dog-bone specimen is similar to that of the cylindrical splitting tensile strength [79]. Some researchers have also found that when 0.5 vol.% macro-straight steel fiber ($l_{f}$/$d_{f}$ = 30/0.8) was added, the splitting tensile strength decreased slightly, which was due to matrix defects in the UHPC due to the uneven distribution of macro-fibers and insufficient matrix fluidity [68]. Gesoglu et al. [44] found that when the fiber content is too low (≤0.5 vol.%), the fiber shape has no effect on the increase in the splitting strength. Jin et al. [46] found that macro hooked-end fibers greatly improved the splitting tensile strength of the UHPC. To sum up, when the fiber content is >0.5 vol.%, regardless of the fiber type, the splitting tensile strength is always positively correlated with the fiber content [44].

#### 4.3.3. Effect of Fiber Length and Hybrids on Splitting Tensile Strength

In the collected papers on splitting tests, most of the researchers believe that the splitting tensile strength is positively related to the fiber length [53,77,81]. However, Abbas et al. [41] found that shorter fibers ($l_{f}$ = 8 mm) are more conducive to improving strength than medium–long fibers. This seems to be related to the shape of the specimen used. In a cylinder, a UHPC with short fibers is better for increasing the splitting strength, while in a prism, a UHPC with medium-length fibers is more beneficial for increasing the splitting strength. Gesoglu et al. [44] found that short, straight fibers ($l_{f}$ = 6 mm) improve strength better than ultra-hooked-end fibers ($l_{f}$ = 30 mm). One possible explanation is that short fibers are better oriented and more numerous relative to ultra-long fibers.

Mizani et al. [68] believed that a 1:1 mixture of long and short steel fibers improved the splitting strength better than a single, long steel fiber. Some researchers have thought that the splitting strength of a long and medium straight fiber hybrid is better than that of a medium and short fiber hybrid or a long and short fiber hybrid [77,81]. In summary, whether the hybridization of steel fibers of different lengths is beneficial to improving the splitting tensile strength of UHPC depends on the synergistic effect between the hybrid fibers.

#### 4.3.4. Empirical Formulas for Splitting Tensile Strength

Table 8 summarizes the empirical prediction formulas for UHPC splitting tensile strength. Research on the prediction formulas has mainly focused on the quadratic equations of fiber content [46], matrix splitting strength, fiber reinforcement coefficient, and matrix compressive strength [82]. In addition, some researchers have studied the influence of temperature and the fiber volume fraction on the splitting tensile strength, and have described the relationship with a cubic function, as shown in Equation (30) [76]. This shows that the steel fibers also have a certain impact on the splitting tensile strength at high temperatures. However, some researchers also believe that the relationship between them is a linear function, as shown in Equation (31) [85].

(30) $$f_{ft,T}=f_{ft}\left[5.13{\left(\frac{T}{1000}\right)}^{3}-8.01{\left(\frac{T}{1000}\right)}^{2}+2.08\frac{T}{1000}+0.9\right],20\;℃\leq T\leq 800\;℃$$

(31) $$f_{ft,T}=f_{ft}\left(1.022-9.21\times 10^{-4}T\right),20\;℃\leq T\leq 900\;℃$$

where *T* is the temperature in degree Celsius.

### 4.4. DIC Application to Tensile Properties of UHPC

#### 4.4.1. The Basic Principle of DIC

**Figure 14:**
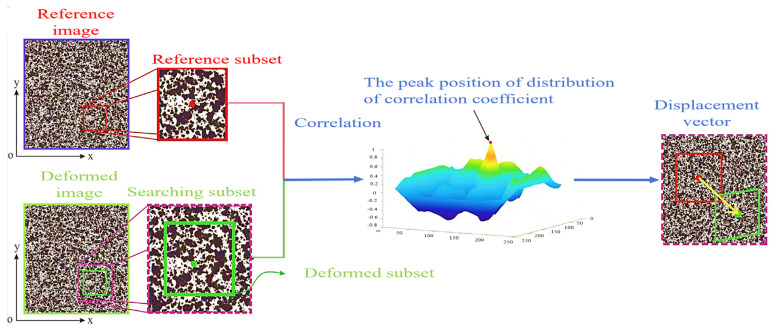
The principle of DIC [128].

**Figure 15:**
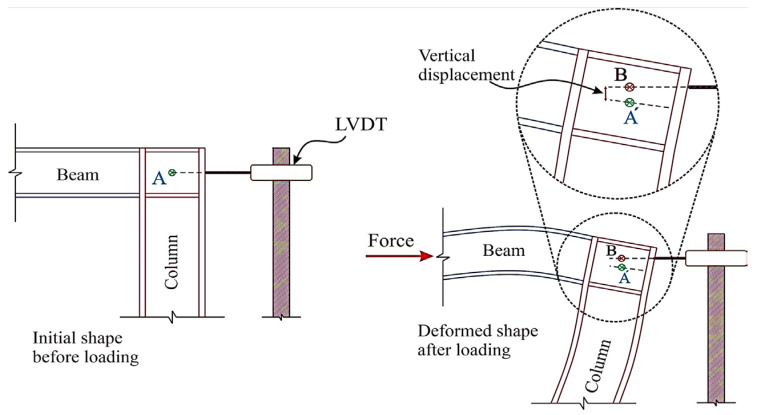
LVDT cannot capture deformation under horizontal and vertical displacement simultaneously [128].

As mentioned before, direct tensile tests are difficult to use to detect the expansion of main cracks using traditional methods. Bending tensile tests often use notched specimens to predetermine the location of crack initiation, and are not suitable for detecting multiple cracks. DIC is a nondestructive and non-contact optical, full-field deformation test [42,70,77,79,81,86,91]. By spraying black and white spots on the surface of the UHPC specimen, a high-definition camera and Vic-Snap software are used to collect the image data. The region to be analyzed is then selected using Vic-2D software [42]. The displacement and deformation are reflected by comparing the unchanged points (x, y) of the image subset to ($x_{1}$, $y_{1}$) in the deformed state (in Figure 14). The horizontal $U\left(x,y\right)$ and vertical $V\left(x,y\right)$ displacement fields are calculated by Equations (34) and (35) [42,91]. The Lagrangian strain field is derived from the displacement field, so as to determine the local cracking zone of the UHPC. Because of the anisotropy of the materials and the geometric shape that affects the shrinkage distribution, DIC can measure the non-uniform surface displacement, which cannot be detected by traditional LVDT [79]. Figure 15 is a schematic diagram of the inability of LVDT to capture the horizontal and vertical deformations at the same time [84]. Therefore, it is significant that DIC technology can be used to study the tensile post-crack curve of UHPC.

(34) $$U\left(x,y\right)=x_{1}-x$$

(35) $$V\left(x,y\right)=y_{1}-y$$

#### 4.4.2. The Role of DIC in the Tensile Properties of UHPC

**Figure 16:**
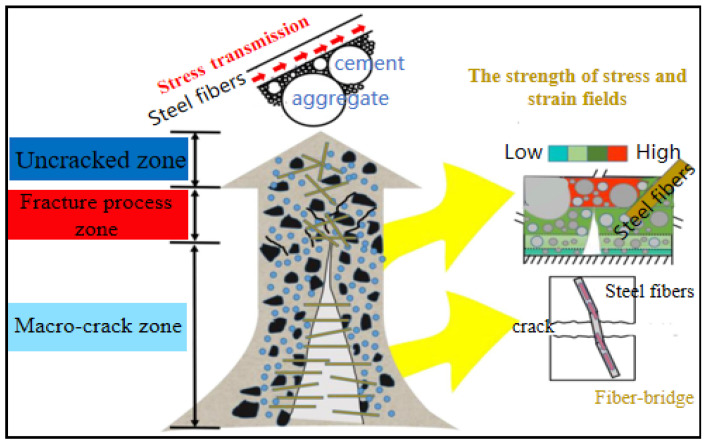
Crack propagation behavior in different regions of UHPC [86].

According to previous studies, the tensile properties of UHPC are closely related to the crack propagation process, especially in the post-crack phase [60,65,129]. Figure 16 depicts the different regions of crack growth behavior in UHPC. The cracking of UHPC beams under a flexural load is divided into three regions: the uncracked zone, fracture process zone, and macro-crack zone [86]. Some researchers believe that there is a micro-fracture zone in the fracture process zone and macro-crack zone, which are called the location zone [70,79]. The crack widths in the fracture process zone and the beginnings of the macro-crack zone are 0.022 mm and 0.05 mm, respectively [70,79,86]. As can be seen from Table 9, DIC is mainly used to characterize the crack propagation mechanism with respect to the tensile properties. Quantifying the strain field and displacement field around the crack, the crack opening displacement of the UHPC, and the deflection of the beam at the crack help to verify the measurement results of the LVDT. Arora et al. [42] presented the effect of the UHPC composition on crack propagation through the combination of mechanical testing and DIC, to better understand the relationship between the material design and performance characteristics of interest. Karim et al. [70] used DIC to obtain first-hand information about the width and depth of the cracks observed in the flexural members, thus, evaluating the influence of steel fibers on crack propagation. Niu et al. [77] used DIC to determine the strain of the first-cracking and the rate of crack propagation. Some researchers have used DIC to analyze the crack shape and length under flexural tensile strength. These show that DIC is widely used to analyze the time evolution of fracture opening displacement, fracture length, and the local strain field [81,86,130]. At the same time, DIC effectively avoids the complex problem of traditional strain gauge arrangement and is conducive to studying the contribution of different steel fibers to crack growth.

## 5. Conclusions

To promote the widespread application of UHPC and to comprehensively understand the tensile properties of steel fibers in UHPC, through reviewing and discussing the collected literature, we can draw the following conclusions:

(1) The standard commonly used for flexural test is ASTMC1609, and the standard commonly used for splitting test is ATSMC496. These standards come from fiber concrete standards and ordinary concrete standards, respectively, and most of them do not refer to the relevant standards for the direct tension test.
(2) In the study of the tensile properties of UHPC, deformed steel fibers ($l_{f}$/$d_{f}$ = 30/0.3) and straight steel fibers ($l_{f}$/$d_{f}$ = 13/0.2) are commonly used. Usually, the tensile strength of the steel fibers is greater than 2000 MPa, thus, avoiding the accidental fracture of the steel fiber.
(3) Whether it is the direct tensile or indirect tensile test, the tensile strength is always proportional to the steel fiber content, and the optimal fiber content seems to be different for different tensile strength test methods. This is related to the shape and size of the specimen and the fiber type. The improvement in the tensile strength of deformed steel fibers is not always better than that of straight steel fibers, which also depends on the size of the steel fibers and the material composition of the UHPC.
(4) Appropriately increasing the length of the steel fibers will help improve the tensile strength. The optimal fiber length is 13~20 mm, which is also related to the orientation of the fibers. The improvement in the tensile strength by hybrid steel fibers is uncertain, and it also depends on the effectiveness of the synergistic effect of the different fibers. It is generally believed that hybrid microfibers and macro steel fibers contribute to the improvement of the tensile strength.
(5) Regarding the effect of steel fibers on the different tensile tests, the relationship between them is complex and nonlinear. Affected by the specimen size effect and cross-sectional stress gradient, the bending tensile test often obtains a tensile strength greater than the actual tensile strength of the UHPC. Direct tension can more intuitively observe the hardening behavior of the UHPC, so it is recommended to use direct tension testing to test the tensile strength of the UHPC.
(6) DIC is promising for replacing traditional strain gauges and displacement gauges. At the same time, the use of DIC helps to evaluate the contribution of the steel fiber type to limiting the crack propagation of UHPC and deepens the understanding of tensile properties. It deserves further attention.

Although a large amount of research has been carried out on the tensile properties of UHPC with steel fibers, there are still many key issues that need to be determined and solved.

(1) Although a large amount of research has been conducted on hybrid steel fibers, the synergistic effect of the different types of deformed steel fibers is not sufficiently understood, which is not conducive for further optimizing the mechanical properties and popularization of UHPC. This deserves further attention and optimization.
(2) At present, there are still conflicting view about the improvement of tensile strength by steel fibers, partly due to the distribution and orientation of steel fibers. Although there have been some studies on this, most of them are based on straight steel fibers, and the predictions of the relevant mechanical models are mostly dependent on experimental results. Because of the different test standards and methods, they are not universally applicable. Therefore, a large amount of research must be conducted in order to obtain a constitutive model of general significance.

## Figures and Tables

**Table 1:** Criteria for inclusion and exclusion of papers.

Inclusion	Exclusion
(a) Steel fiber reinforcement.	(a) Non-steel fiber reinforcement.
(b) English language.	(b) Non-English language.
(c) Tensile strength (direct strength, flexural strength, spilling strength).	(c) Impact, blast, shear, fatigue.
(d) Journal or conference papers.	(d) Numerical or analytical studies.
(e) Experimental research papers.	(e) Structural members (beams, slabs, pillar).

**Table 2:** Included research papers.

Ref.	Test Properties	Fiber Combination	Fiber Types ( $l_{f}$$/d_{f}$) and Volume Fraction *	Fiber Tensile Strength [MPa]
[41]	Flexural strength, splitting strength	Single	SSF (8/0.2, 12/0.2, 16/0.2); 0%, 1%, 3%, 6%.	>2850
[42]	Flexural strength	Single	SSF (13/0.2); 0%, 1%, 3%.	1900
[43]	Direct tensile strength	Single + Hybrid	SSF (13/0.2, 30/0.3), HSF (30/0.375), TSF (30/0.3); 2%.	2428~2900
[44]	Flexural strength, splitting strength	Single	SSF (6/0.16), HSF (30/0.55); 0%, 0.25%, 0.5%, 0.75%, 1%, 1.5%, 2%.	1345~2250
[45]	Direct tensile strength	Single	SSF (13/0.2); 0%, 2%.	-
[46]	Flexural strength, splitting strength	Single	HSF (30/0.6); 0%, 1%, 2%, 3%.	1100
[47]	Flexural strength	Single + Hybrid	SSF (13/0.2, 30/0.3), HSF (30/0.375, 62/0.775), TSF (30/0.3); 1%, 1.5%, 2%, 2.5%.	1891~2788
[40]	Direct tensile strength	Single	SSF (9/0.15, 13/0.175, 20/0.25); 0%, 1.5%, 3%.	2500
[48]	Direct tensile strength	Single	SSF (13/0.2), SPSF (13/0.2), HSF (13/0.2, 30/0.6); 0%, 1%, 1.75%, 2.5%.	1890~2940
[49]	Flexural strength	Single + Hybrid	SSF (13/0.22), HSF (13/0.22); 2.5%.	2850
[50]	Flexural strength, direct tensile strength	Single + Hybrid	SSF (13/0.2), HSF (30/0.5); 0%, 1%, 2%, 3%, 4%, 5%.	1900
[51]	Flexural strength	Single	SSF (13/0.2, 19.5/0.2, 30/0.3); 0.5%, 1%, 1.5%, 2%.	2580~2788
[52]	Direct tensile strength	Single + Hybrid	SSF (13/0.2, 30/0.3), HSF (30/0.375, 62/0.775), TSF (30/0.3); 1%, 1.5%, 2%, 2.5%.	1891~2788
[53]	Flexural strength, splitting strength	Single	SSF (6/0.16, 13/0.16); 0%, 2%, 2.5%.	2000
[54]	Flexural strength	Single + Hybrid	SSF (13/0.2, 16.3/0.2, 19.5/0.2), HSF (30/0.375); 2%.	2311~2700
[55]	Flexural strength	Single + Hybrid	SSF (13/0.2, 16.3/0.2, 19.5/0.2); 1.5%, 2%.	--
[56]	Flexural strength	Single	SSF (13/0.2); 0%, 1%, 2%, 3%.	2850
[57]	Direct tensile strength	Single	SSF (13/0.2), HSF (30/0.38), TSF (18/0.3); 1.5%, 2%, 2.5%, 3%.	2100~2900
[58]	Direct tensile strength	Single	SSF (13/0.2), HSF (30/0.38), TSF (30/0.3); 1%, 1.5%, 2%, 2.5%.	2100~3100
[59]	Flexural strength	Single	SSF (13/0.2), HSF (13/0.2), CSF (13/0.2); 0%, 2%.	2800
[60]	Flexural strength	Single + Hybrid	SSF (6/0.2, 13/0.2); 0%, 2%.	2800
[61]	Flexural strength	Single	SSF (13/0.2), HSF (13/0.2), CSF (13/0.2); 0%, 1%, 2%, 3%.	2800
[62]	Flexural strength	Single	SSF (13/0.2, 16.3/0.2, 19.5/0.2); 2%.	2500
[63]	Flexural strength	Single + Hybrid	SSF (13/0.2, 19.5/0.2), HSF (30/0.38), TSF (30/0.3); 2%.	2428~2788
[64]	Direct tensile strength	Single	SSF (13/0.2), HSF (30/0.375, 25/0.375), TSF (30/0.3); 2%.	2428~2900
[65]	Flexural strength	Single	SSF (13/0.2, 19.5/0.2, 30/0.3), HSF (30/0.38), TSF (30/0.3); 0.5%, 1%, 1.5%, 2%.	2428~2788
[66]	Flexural strength	Single + Hybrid	SSF (13/0.2, 19.5/0.2, 30/0.2); 0%, 0.5%, 1%, 1.5%, 2%.	2500~2788
[67]	Flexural strength	Single + Hybrid	SSF (13/0.2), HSF (20/0.25, 20/0.35); 2%.	2810~2940
[68]	Flexural strength, splitting strength	Single + Hybrid	SSF (30/0.8, 13/0.2); 0%, 0.5%, 1.5%.	700~2500
[69]	Flexural strength, splitting strength	Single	SSF (15/0.6); 0%, 2%.	1700
[70]	Flexural strength	Single + Hybrid	SSF (13/0.2), TSF (25/0.5), HSF (34/0.54); 1%, 1.5%, 2%, 2.5%, 3%.	1100~2000
[71]	Flexural strength	Single	SSF (6/0.16, 13/0.2, 20/0.2); 2%.	2850
[72]	Flexural strength	Single	SSF (6/0.16, 13/0.2, 20/0.2); 1%, 2%, 3%.	2850
[73]	Flexural strength	Single + Hybrid	SSF (13/0.2), HSF (25/0.2); 2%.	2500
[74]	Flexural strength	Single + Hybrid	SSF (6/0.2, 10/0.2, 15/0.2); 2.5%.	>2850
[75]	Direct tensile strength	Single	SSF (13/0.2); 0%,1.3%, 1.8%.	2850
[76]	Splitting strength	Single	SSF (13/0.22); 2%.	>2850
[77]	Flexural strength, splitting strength	Single + Hybrid	SSF (6/0.2, 13/0.2, 20/0.2); 0%, 2%.	-
[78]	Flexural strength, splitting strength	Single	SSF (35/0.9); 0%, 2%.	1250
[79]	Flexural strength, splitting strength, direct tensile strength	Single	SSF (13/0.2), HSF (13/0.22, 16/0.22, 16/0.25); 0%, 1%, 2%, 3%.	2500~2800
[80]	Direct tensile strength	Single	SSF (13/0.2); 0%, 0.5%, 1%, 1.5%, 2%, 2.5%.	2850
[81]	Flexural strength, splitting strength	Single + Hybrid	SSF (6/0.2, 13/0.2, 20/0.2); 0%, 2%.	2500~2788
[82]	Flexural strength, splitting strength	Single	SSF (12/0.2); 0%, 0.5%, 1%, 1.5%, 2%, 2.5%, 3%.	2850
[83]	Flexural strength	Single	SSF (13/0.2); 0%, 1%, 2%, 2.5%.	2850
[84]	Flexural strength, direct tensile strength	Hybrid	SSF (13/0.16), HSF (30/0.76); 2%.	1900~2700
[85]	Flexural strength, splitting strength	Single	SSF (13/0.22); 2%.	2850
[86]	Flexural strength, splitting strength	Single	SSF (13/0.2); 0%, 0.5%, 1%, 1.5%, 2%.	-
[87]	Flexural strength, direct tensile strength	Single	SSF (13/0.2), TSF (13/0.5); 1%, 1.5%, 2%, 2.5%, 3%.	-
[88]	Direct tensile strength	Single	SSF (20/0.3, 13/0.175), HSF (35/0.75, 35/0.55, 30/0.35); 0%, 1.5%, 2%, 3%, 4%, 5%.	1000~1250
[89]	Direct tensile strength	Single	SSF (13/0.4); 0.75%, 1%, 1.5%, 2%.	-
[90]	Flexural strength	Single	SSF (13/0.2); 2%.	1900
[91]	Flexural strength	Single	SSF (13/0.2); 0%, 1%, 3%.	1900
[92]	Flexural strength, splitting strength	Single	SSF (13/0.2); 1.5%, 2%, 2.5%, 3%.	≥2800
[93]	Direct tensile strength	Single + Hybrid	SSF (7/0.18), HSF (13/0.22, 35/0.58); 0%, 1%, 2%, 3%, 4%, 5%.	≥2850
[94]	Flexural strength	Single	SSF (13/0.25, 17/0.25, 13/0.2, 17/0.2); 1%, 2.5%.	1980~2000

* SSF (straight steel fibers); HSF (hooked-end steel fibers); TSF (twisted steel fibers); SPSF (spiral steel fibers).

**Table 3:** Direct tension test setups.

Schematic Diagram of Sample Shape	Cross-Sectional Testing Area [mm^2^]	Test Standard	Loading Rate	Ref.
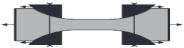	30 × 13	According to JSCE [99].	0.4 mm/min.	[43]
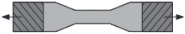	26 × 50	No standard.	0.4 mm/min.	[45]
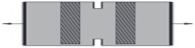	40 × 40 (notched)	No standard.	0.6 mm/min. and 0.3 mm/min.	[40]
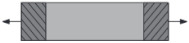	100 × 100	No standard.	0.05 mm/min.	[48]
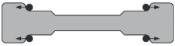	50 × 25	No standard.	0.05 mm/min.	[50]
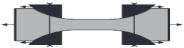	50 × 100	No standard.	0.4 mm/min.	[52]
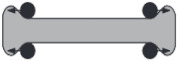	25 × 25	According to AASHTO T 132–87 [96].	0.6 mm/min.	[57]
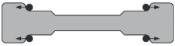	50.8 × 25.4	No standard.	--	[58]
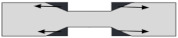	30 × 13	According to JSCE [99].	0.4 mm/min.	[64]
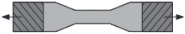	50 × 100	According to GB/T 50081-2019 [97] and T/CBMF 37-2018 [100].	0.15 mm/min.	[75]
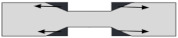	30 × 13	According to JSCE [99].	0.5 mm/min.	[79]
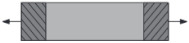	100 × 100	According to NF P 18-710 [101].	0.5 mm/min.	
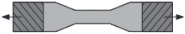	50 × 100	No standard.	0.2 mm/min.	[80]
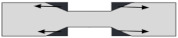	40 × 40	No standard.	0.1 mm/min.	[84]
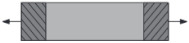	51 × 51	According to FHWA [98].	0.05 mm/min.	[87]
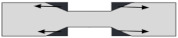	40 × -	No standard.	0.1 mm/min.	[88]
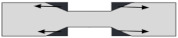	50 × 50	No standard.	0.4 mm/min.	[89]
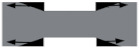	60 × 130	No standard.	0.05 mm/min.	[93]

**Table 4:** Empirical formula, constitutive model, and prediction formula for direct tensile strength of UHPC.

Ref.	Prediction Formula	Fiber Shape	Fiber Volume Content	Eq.
[57]	$f_{t}=-0.9{V_{f}}^{2}+9V_{f}$	Straight	1.5~3%	(4)
		Hooked end		
		Twisted		
[106]	$f_{t}=\lambda \times \tau V_{f}\frac{l_{f}}{d_{f}}$	-	-	(5)
[40]	$f_{t}=4.82\ln K+9.08$ $K=V_{f}\frac{l_{f}}{d_{f}}$	Straight	1.5~3%	(6)
[103]	$\sigma =\left\{\begin{array}{c} \frac{\sigma_{pc}}{\varepsilon_{ca}}\varepsilon \;\;\;\;,\;\;\;\;\;0\leq \varepsilon \leq \varepsilon_{ca}\;\\ \sigma_{pc}\;\;\;\;,\;\varepsilon_{ca}\leq \varepsilon \leq \;\varepsilon_{pc}\;\;\;\;\;\;\;\;\\ \sigma_{pc}\frac{1}{{\left(1+w/w_{p}\right)}^{p}}\;,\;0<w \end{array}\right.$	Straight	2~3.5%	(7)
		Hooked end		
	σ=σpcεcaε , 0≤ε≤εca σpc , εca≤ε≤ εpc σpc − σpc − σw1w1w ,wpc < w ≤ w1σw1+σw1 − σw2w1 − w2w1 − w ,w1 < w ≤ w2	Hooked end	2~3.5%	(8)
		Straight		
[104]	$y=\left\{\begin{array}{c} 1.17x-0.65x^{2}-0.83x^{3}\;\;\;,\;0\leq x<1\\ \frac{x}{5.5{\left(x\;-\;1\right)}^{2.2}\;+\;x}\;\;\;,\;\;1\leq x \end{array}\right.$ $x=\frac{\varepsilon }{\varepsilon_{pc}};y=\frac{\sigma }{\sigma_{pc}}$	Straight	2%	(9)
[79]	σ=εεccσcc , 0≤ε≤εccε − εccεu1 − εccσpc − σcc + σcc, εcc ≤ ε ≤ εu1σpc, εu1 ≤ ε ≤ εu2σpc11 + w/wpp , 0<w	Straight	2%	(10)
		Hooked end	0~3%	
[105]	$\sigma =\left\{\begin{array}{c} \frac{\varepsilon }{\varepsilon_{cc}}\sigma_{cc}\;,\;\;\;\;\;\;\;\;\;\;\;\;\;\;\;\;\;\;\;\;\;\;\;\;\;\;0\leq \varepsilon \leq \varepsilon_{cc}\\ \frac{\varepsilon -\varepsilon_{cc}}{\varepsilon_{pc}-\varepsilon_{cc}}\left(\sigma_{pc}-\sigma_{cc}\right)+\sigma_{cc}\;,\;\varepsilon_{cc}\leq \varepsilon \;\leq \;\varepsilon_{pc}\\ \left(1-\frac{\varepsilon -\varepsilon_{pc}}{\varepsilon_{u}-\varepsilon_{pc}}\right)\sigma_{pc}\;\;\;\;\;\;\;\;\;\;\;\;\;\;\;\;,\;\;\;\;\;\;\;\;\varepsilon_{pc}<\varepsilon \; \end{array}\right.$	-	-	(11)
[80]	$f_{t}=\sigma_{m}\left(1+0.382V_{f}\frac{l_{f}}{d_{f}}\right)$	Straight	0.5~2.5%	(12)
	$\sigma =\sigma_{pc}.exp\left[\frac{\ln0.3}{4.129V_{f}\frac{l_{f}}{d_{f}}\;+\;0.591}\omega^{0.451{\left(V_{f}\frac{l_{f}}{d_{f}}\right)}^{0.899}}\right],\omega >0$			(13)
[84]	$y=\frac{a\;+\;bx}{1\;+\;cx\;+\;dx^{2}}$ $x=\frac{\varepsilon }{\varepsilon_{pc}};\;y=\frac{\sigma }{\sigma_{pc}}$	Straight and hooked-end hybrid	2%	(14)
[88]	$f_{t}=3.5119-0.0178\times \frac{l_{f}}{d_{f}}+1.876\times V_{f}$	Straight	0~4%	(15)
	$f_{t}=\sigma_{m}+0.0028\times RI$ $RI=\frac{\rho_{f}}{\rho_{c}}\times V_{f}$	Hooked end	0~4%	(16)

**Table 5:** Flexural tensile strength test setups.

Schematic Diagram of Sample Shape	Test Standard	Loading Rate	Specimen Size [mm^3^]	Ref.
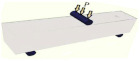	According to ASTM C1609 [108].	0.05 mm/min.	100 × 100 × 400	[41]
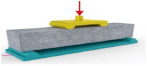	According to ASTM C1609 [108].	0.5 mm/min.	100 × 100 × 457	[42]
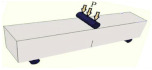	According to RILEM 50-FMC/198 [111].	0.02 mm/min.	70 × 70 × 280 (notched)	[44]
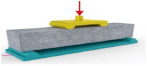	According to CECS 13:2009 [112].	--	100 × 100 × 400	[46]
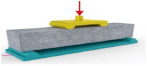	According to ASTM C1018-97 [113] and ASTM C 1609 [108].	0.4 mm/min.	100 × 100 × 350	[47]
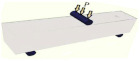	According to BS EN 196-1 (CEN 2005) [114].	--	40 × 40 × 160	[49]
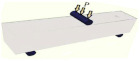	According to ASTM C1609 [108].	--	76.2 × 76.2 × 304.8	[50]
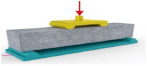	According to ASTM C1609 [108].	0.4 mm/min.	100 × 100 × 400	[51]
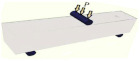	According to ASTM C1609 (ASTM, 2006) [108].	--	70 × 70 × 350 (notched)	[53]
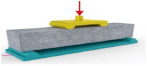	No standard.		--	[54]
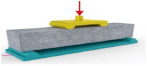	No standard.	0.2 mm/min.	--	[55]
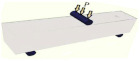	According to GB/T 17671-1999 [107].	--	40 × 40 × 160	[56]
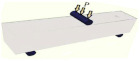	No standard.	1 mm/min.	40 × 40 × 160	[59]
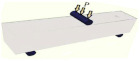	According to GB/T 17671-1999 [107].		40 × 40 × 160	[60]
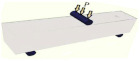	No standard.	0.2 mm/min.	40 × 40 × 160	[61]
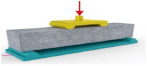	According to ASTM C 1609/C 1609M [108].	0.4 mm/min.	100 × 100 × 400	[62]
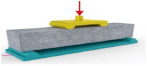	According to ASTM C1609 [108].	0.4 mm/min.	100 × 100 × 400	[63]
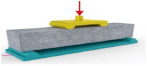	According to ASTM C1609 [108].	0.4 mm/min.	100 × 100 × 400	[65]
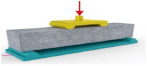	According to ASTM C1609 [108].	0.4 mm/min.	100 × 100 × 400	[66]
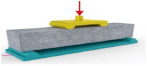	According to ASTM C1609 [108].	0.1 mm/min.	100 × 100 × 400	[67]
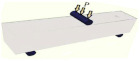	According to ASTM C1609 [108].	0.05 mm/min.	100 × 100 × 450	[68]
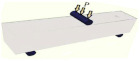	According to ASTM C293 [115].	0.05 MPa/min.	100 × 100 × 500	[69]
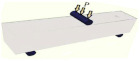	According to ASTM C1609 [108].	1.83 mm/min.	100 × 100 × 350	[70]
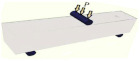	No standard.	0.4 mm/min.	70 × 70 × 230	[71]
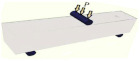	No standard.	0.4 mm/min.	70 × 70 × 230	[72]
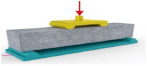	According to French interim UHPC guideline annex [116].	0.5 mm/min.	70 × 70 × 280	[73]
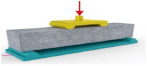	No standard.	0.1 kN/s and 0.3 mm/min.	100 × 100 × 400	[74]
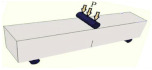	No standard.	0.1 kN/s and 0.3 mm/min.	100 × 100 × 400 (notched)	
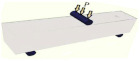	According to ASTM C1609 [108].	0.2 mm/min.	100 × 100 × 400	[77]
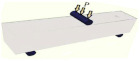	According to ASTM C1609 [108].		100 × 100 × 350	[78]
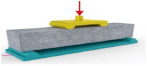	According to NF P 18-710 [101].	0.2 mm/min.	100 × 100 × 400	[79]
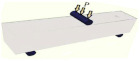	According to ASTM C1609 [108].	0.2 mm/min.	100 × 100 × 400	[81]
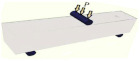	According to ASTM C 348 [117].		40 × 40 × 160	[82]
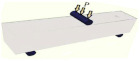	No standard.	0.4 mm/min.	70 × 70 × 230	[83]
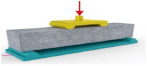	According to ASTM C1609/C1609M [108].	--	100 × 100 × 500	[84]
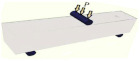	According to GB/T 50081 [118].	0.05 mm/min.	70.7 × 70.7 × 220	[85]
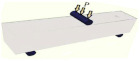	According to ASTM C1609 [108].	0.05 mm/min.	100 × 100 × 400	[86]
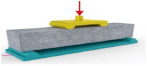	According to ASTM C1609 [108] and ASTM C1856 [119].	--	102 × 102 × 356	[87]
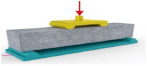	According to ASTM C1609 [108].	0.1 mm/min.	75 × 75 × 305	[90]
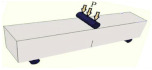	According to ASTM C1609 [108].	0.5 mm/min.	64 × 51 × 381	[91]
			100 × 100 × 457	
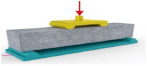	According to CECS (2013) [112].	--	100 × 100 × 400	[92]
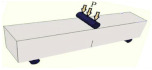			100 × 100 × 400 (notched)	
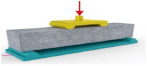	According to DIN EN 12390-5 [120].	0.6 mm/min.	40 × 40 × 160	[94]

**Table 6:** UHPC flexural strength empirical prediction formula.

Ref.	Empirical Formula	Fiber Type	Fiber Content	Eq.
[46]	$f_{ff}=f_{fm}\left(1+1.25V_{f}\frac{l_{f}}{d_{f}}\right)$	Hooked end	0~3%	(21)
[59]	$f_{ff}=f_{fm}\left(1-V_{f}\right)+\alpha \eta_{\theta }\tau V_{f}\frac{l_{f}}{d_{f}}$	Straight	2%	(22)
		Hooked end		
		Corrugated		
[66]	$f_{ff}=\left\{\begin{array}{c} f_{fm}\;\;\;\;\;\;\;\;\;\;\;\;\;\;\;\;\;\;\;\;\;\;\;\;\;\;\;\;\;\;\;\;\;\;\;\;\;\;\;\;,V_{f}\frac{l_{f}}{d_{f}}\leq 0.5\\ {1.471f}_{fm}\ln\left(V_{f}\frac{l_{f}}{d_{f}}\right)+2.02f_{fm},V_{f}\frac{l_{f}}{d_{f}}>0.5 \end{array}\right.$	Straight	0~2%	(23)
[71]	$f_{ff}=24.42+18.31\frac{l_{f}}{H}$	Straight	2%	(24)
[72]	$f_{ff}=\frac{4}{3}f_{fm}\left(1-V_{f}\right)+\beta \eta_{\theta }V_{f}\left(\sum c_{i}\tau_{i}\frac{l_{fi}}{d_{fi}}\right)$	Straight	1~3%	(25)
[73]	$f_{ff}=28.7+0.099FP$ *	Straight	2%	(26)
		Hooked end		
[82]	$f_{ff}=1.31{V_{f}}^{2}+2.37V_{f}+10.6$	Straight	0~3%	(27)
[83]	$f_{ff}=7.576+6.244\lambda b/w$ $\lambda =\alpha \eta_{\theta }V_{f}$	Straight	0~2.5%	(28)

* *FP* = hooked-end fiber content = 100 − straight fibers content; $f_{fm}$ is the flexural strength of the matrix.

**Table 7:** Splitting tensile strength test setups.

Author(s), (Year)	Test Standard	Loading Rate	Shape and Size [mm^3^]	Ref.
Abbas et al. (2015)	According to ASTM C496/C496M [127].	0.025 mm/min.	Cylinder: 75 × 150	[41]
Gesoglu et al. (2016)	According to ASTM C496 [127].	--	Cubes: 100 × 100 × 100	[44]
Jin and Zhang et al. (2018)	According to CECS 13:2009 [112].	--	Cubes: 100 × 100 × 100	[46]
Prem et al. (2015)	According to ASTM C1609 [108].	0.03 mm/min.	Prisms: 70 × 70 × 350 (notched)	[53]
Mizani and Sadeghi et al. (2022)	According to ASTM C496 [127].	1 MPa/min.	Cylinder: 150 × 300	[68]
Raza et al. (2021)	According to ASTM C496 [127].	--	Cylinder: 100 × 200	[69]
Mao et al. (2021)	According to GB/T 50081–2019 [97].	0.08 MPa/s.	Cubes: 100 × 100 × 100	[76]
Niu et al. (2021)	No standard.	1.2 MPa/s.	Cubes: 100 × 100 × 100	[77]
Raza et al. (2021)	According to ASTM C496 [127].	--	Cylinder: 100 × 200	[78]
Fang et al. (2022)	According to ASTM C496 [127].	1 kN/s.	Cylinder: 100 × 200	[79]
			Cylinder: 150 × 300	
Jiao et al. (2022)	According to ASTM C496 [127].	1.2 MPa/s.	Cubes: 100 × 100 × 100	[81]
Ashkezari et al. (2020)	According to ASTM C 496 [127].	--	Cylinder: 150 × 300	[82]
Abid et al. (2019)	According to GB/T 50081 [118].	0.05 mm/min.	Cubes: 70.7 × 70.7 × 70.7	[85]
Meng et al. (2022)	According to ASTM C1069 [108].	0.12 MPa/s.	Cubes: 100 × 100 × 100	[86]
Wang et al. (2022)	According to CECS (2013) [112].	--	Cubes: 100 × 100 × 100	[92]

**Table 8:** Empirical formula for splitting tensile strength of UHPC.

Ref	Empirical Formula	Fiber Type	Fiber Content	Eq.
[46]	$f_{ft}=f_{mt}\left(1+1.03V_{f}\frac{l_{f}}{d_{f}}\right)$ *	Hooked end	0~3%	(32)
[82]	$f_{ft}=-1.22{V_{f}}^{2}+7.27V_{f}+4.98$	Straight	0~3%	(33)
	$f_{ft}=\left\{\begin{array}{c} 3.86\sqrt{f_{c}}-28.4\\ 0.133\left[f_{c}+10\sqrt{V_{f}\frac{l_{f}}{d_{f}}b_{f}}\left(3-\frac{20}{f_{c}}\right)\right]-5.01 \end{array}\right.$ $f_{c}=73.5+37.14V_{f}-6.06{V_{f}}^{2}$			

* $f_{mt}$ is the splitting strength of the matrix.

**Table 9:** Application of DIC to steel fiber-reinforced UHPC.

Ref.	Fiber Combination	Fiber Shape	Application of DIC to UHPC
[42]	Single	Straight	(a) Characterize the main cracks and secondary cracks of the UHPC. (b) Analysis of the strain field at different load values.
[70]	Single + Hybrid	Hooked end	(a) Analysis of the strain field representing different deflections. (b) The maximum crack width of the UHPC reinforced with different steel fibers shapes. (c) Quantify the crack width within the depth range of the sample.
		Twisted	
		Straight	
[77]	Single + Hybrid	Straight	(a) Characterize the crack shape and strain distribution under different load values. (b) Quantify the crack width within the depth range of the sample. (c) Quantification of the crack growth rate.
[79]	Single	Straight	(a) Comparison of the strain cloud maps and failure patterns for the different specimens.
		Hooked end	
[81]	Single + Hybrid	Straight	(a) Characterize the crack shape and strain distribution under different load values. (b) Quantify the crack width within the depth range of the sample.
[86]	Single	Straight	(a) Characterization of horizontal displacement field of UHPC in different loading stages. (b) Changes in crack propagation in different loading stages. (c) Displacement of crack opening in different loading stages.
[91]	Single	Straight	(a) Comparison between DIC and traditional LVDT measurement methods. (b) Quantify crack width over a range of specimen heights. (c) Characterize the strain distribution under different load values.
[131]	Single	Straight	(a) Crack propagation in different loading stages. (b) Crack width in the range of sample depth under different loads.

## Data Availability

Data are contained within the article.
